# Entrectinib—A SARS-CoV-2 Inhibitor in Human Lung Tissue (HLT) Cells

**DOI:** 10.3390/ijms222413592

**Published:** 2021-12-18

**Authors:** Alejandro Peralta-Garcia, Mariona Torrens-Fontanals, Tomasz Maciej Stepniewski, Judith Grau-Expósito, David Perea, Vikram Ayinampudi, Maria Waldhoer, Mirjam Zimmermann, María J. Buzón, Meritxell Genescà, Jana Selent

**Affiliations:** 1 Research Programme on Biomedical Informatics (GRIB), Department of Experimental and Health Sciences, Hospital del Mar Medical Research Institute (IMIM), Pompeu Fabra University (UPF), 08003 Barcelona, Spain; alejandro.peralta04@estudiant.upf.edu (A.P.-G.); mariona.torrens@upf.edu (M.T.-F.); tm.stepniewski@gmail.com (T.M.S.); 2 InterAx Biotech AG, PARK InnovAARE, 5234 Villigen, Switzerland; ayinampudi@interaxbiotech.com (V.A.); waldhoer@interaxbiotech.com (M.W.); zimmermann@interaxbiotech.com (M.Z.); 3 Infectious Diseases Department, Vall d’Hebron Institut de Recerca (VHIR), Vall d’Hebron Barcelona Hospital Campus, Vall d’Hebron Hospital Universitari, 08035 Barcelona, Spain; judit.grau@vhir.org (J.G.-E.); david.perea@vhir.org (D.P.); mariajose.buzon@vhir.org (M.J.B.); meritxell.genesca@vhir.org (M.G.)

**Keywords:** SARS-CoV-2, COVID-19, drug repurposing, virtual screening, viral cell entry assays

## Abstract

Since the start of the COVID-19 outbreak, pharmaceutical companies and research groups have focused on the development of vaccines and antiviral drugs against SARS-CoV-2. Here, we apply a drug repurposing strategy to identify drug candidates that are able to block the entrance of the virus into human cells. By combining virtual screening with in vitro pseudovirus assays and antiviral assays in Human Lung Tissue (HLT) cells, we identify entrectinib as a potential antiviral drug.

## 1. Introduction

Severe acute respiratory syndrome coronavirus 2 (SARS-CoV-2) is the pathogen that causes the novel coronavirus disease-2019 (COVID-19). First detected in Wuhan, China, in December 2019, it quickly spread across the country and, by 11 March 2020, the World Health Organization (WHO) declared COVID-19 a global pandemic, present in almost every country across the globe. Since then, as of November 2021, 260 million people have been infected, counting 5.2 million deaths worldwide [1].

SARS-CoV-2 belongs to the broad family of coronaviruses, a group of RNA viruses that infect mammals and birds. They are enveloped viruses with a positive-sense single-stranded RNA genome and a nucleocapsid of helical symmetry. In humans and birds, they cause respiratory tract infections that can range from mild to lethal. Particularly, SARS-CoV-2 is a member of the betacoronavirus genus. Some betacoronaviruses, such as HCoV-OC43 and HCoV-HKU1, cause mild to moderate respiratory illnesses such as the common cold (which is also caused by other viruses, predominantly rhinoviruses). However, other betacoronaviruses are responsible for severe respiratory illnesses that can pose a threat to human health, including not only SARS-CoV-2 but also severe acute respiratory syndrome (SARS) and Middle East respiratory syndrome (MERS) coronaviruses, which have resulted in previous epidemics [2,3,4].

Cellular infection with SARS-CoV-2 is initiated by the binding of the spike protein to its cellular receptor angiotensin-converting enzyme 2 (ACE2) [5]. This triggers the endocytosis of virions, with the envelope intact. Once inside the endosomal vesicle, induced lysosomal proteases cleave the spike protein, mediating fusion of the viral envelope with the endosomal membrane [6]. Subsequently, the viral genome is released into the cytosol, where it starts transcription and replication processes [7].

Major pharmaceutical companies have focused on vaccine development as the primary response to the COVID-19 outbreak. At the same time, numerous research groups and pharmaceutical companies are looking for antiviral drugs to be used as non-immunological interventions. Several drugs targeting viral proteins are being used or are under investigation for use against SARS-CoV-2. Some of the main targets are the spike protein, the RNA-dependent RNA polymerase (RdRp or nsp12), and the main protease (Mpro or nsp5). This is the case of remdesivir (RdRp inhibitor) [8], ritonavir, lopinavir, and PF-07321332 (inhibitors of viral proteases) [9,10,11], or plitidepsin (inhibitors of the viral replication) [12]. Particularly, antiviral drugs targeting the spike protein are considered a promising strategy, as they block the host cell recognition and viral entry. For instance, therapeutic antibodies targeting the spike protein have been approved by the Food and Drug Administration (FDA) for the treatment of COVID-19 (e.g., Bamlanivimab + Etesevimab, Casirivimab + Imdevimab) [13]. In addition, drugs that interfere with the host cell lipid metabolism, which is critical for viral infection (e.g., inhibitors of the acid sphingomyelinase activity), are of particular interest [14,15] and have shown favorable results in in vitro [16] and clinical [17] studies.

In this work, we aim to discover antiviral compounds that are able to block SARS-CoV-2 entry into the cell using a drug repurposing strategy that represents a promising approach to find candidates at a lower cost and in a shorter time [18]. For this, we combine virtual screening with cell-based as well as human lung tissue (HLT) assays yielding entrectinib as a potential drug candidate.

## 2. Results

A promising strategy to prevent SARS-CoV-2 infections is to inhibit the initial step of viral entry into the host cell. The SARS-CoV-2 spike protein and in particular the receptor-binding domain (RBD) are crucial for viral attachment to the ACE2 host cell receptor. In a first step, we explored which binding contacts in the RBD–ACE2 interface are most relevant for viral attachment. This information was exploited in a virtual screen for drug candidates that are able to interfere with the viral attachment interface. Promising candidates were validated in cell-based as well as in HLT assays.

### 2.1. Targeting the RBD–ACE2 Interface for a SARS-CoV-2-Specific Antiviral Action

#### 2.1.1. Molecular Dynamics Simulations of the RBD–ACE2 Interface Reveal Contact Hotspots

**Figure 1:**
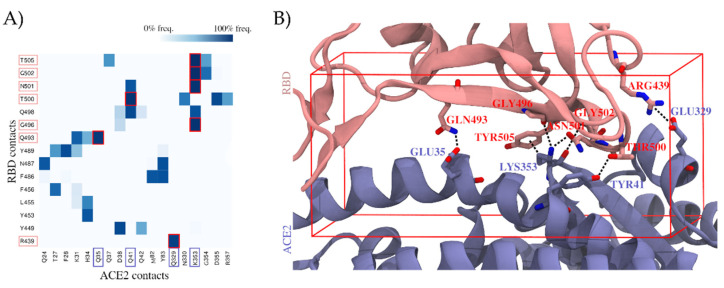
Contribution of individual residues to the stability of the binding complex formed by the spike protein’s receptor-binding domain (RBD) and the angiotensin-converting enzyme 2 (ACE2), computed as contact frequencies. (**A**) Stability of RBD–ACE2 contacts. Heatmap of contact frequencies between RBD and ACE2 residues, where contact frequencies are represented as a color scale from white (0%) to dark blue (100%). In the red square are depicted those contacts that are maintained through >90% of the simulation, and the corresponding RBD and ACE2 residues are highlighted with salmon and blue squares, respectively, in the axis labels. Residues that do not form any interaction with frequency >50% were filtered out. (**B**) Structural mapping of the most stable contacts (contact frequency >90%) between RBD and ACE2. The interface region where these contacts are found was used to guide a virtual screening. The docking box applied in virtual screening is indicated in red.

To identify which residues of the RBD are most relevant for establishing the ACE2 interactions, we explored the RBD–ACE2 interface in long-scale molecular dynamics simulations with 10 μs of simulation time (Figure 1A and Section 4.1 (RBD–ACE2 Interface Examination)). Relevant hotspots were detected by computing the contact frequencies between individual residues. Critical regions with more than 90% contact frequencies were mapped on the RBD–ACE2 interface and involved the following residues: ARG439, GLN493, GLY496, THR500, ASN501, GLY502, and TYR505 (Figure 1B). They represent valuable information to guide the subsequent virtual screen.

#### 2.1.2. Virtual Screening Yields Two Drug Candidates with the Potential to Inhibit SARS-CoV-2 Cell Entry

For our drug repurposing strategy, we created a curated database of 5849 compounds including drugs approved by the FDA and the European Medicines Agency (EMA), as well as known drug metabolites (see Section 4.2 (Database Creation)). The curated database was docked into the RBD–ACE2 interface focusing in particular on regions important for the complex stability determined in the previous step (see docking box in Figure 1B and Section 4.1 (RBD–ACE2 Interface Examination)). We carried out two individual screens targeting the binding interface of the RBD and the ACE2.

Among the top hits of both screenings, we manually selected five promising candidates to be tested in a cell-based assay (Table 1 and Section 4.3 (Molecular Docking)). In addition, we included four more compounds that had been related to antiviral SARS-CoV-2 activity. (i) Ivermectin, with a proven antiviral effect, was proposed to bind the RBD [19], as well as (ii) argatroban and (iii) otamixaban—two compounds that have been suggested to inhibit viral cell entry in a computational study by interfering with the transmembrane protease TMPRSS2a [20]. Ultimately, we included (iv) apilimod as a positive control—a PIKfyve inhibitor [21] that has been demonstrated to block the viral entrance in vitro [18] (Table 1), likely by disrupting host endocytosis by inhibiting the early endosome to the late endosome pathway [22]. However, its value as a drug candidate against COVID-19 has been questioned, as the inhibited proteases are also critical for efficient antiviral immune responses, which would be counterproductive for the treatment [23].

### 2.2. Proof of Concept

#### 2.2.1. Pseudovirus Assay Confirms Anti-SARS-CoV-2 Activity for Entrectinib and Nilotinib

**Figure 2:**
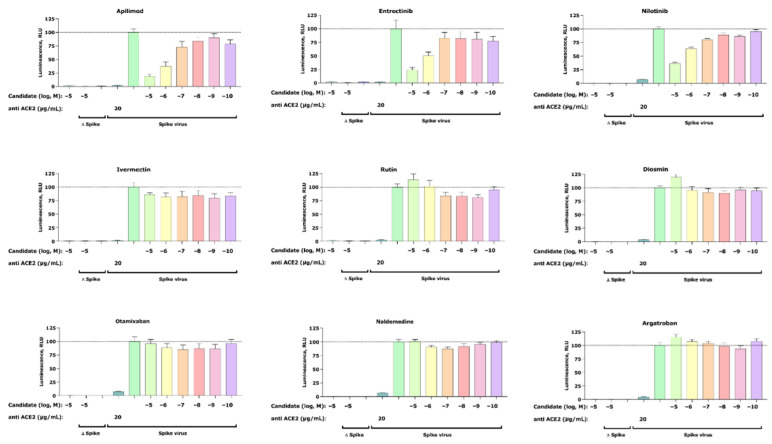
Inhibition of the cell entry of SARS-CoV-2 pseudotyped particles. The 293 T cells transiently expressing human ACE2 and TMPRSS2a were pre-treated with the selected compounds at the concentrations indicated for 3 h or with 20 μg/mL of the human anti-ACE2 antibody (positive control) before they were inoculated with SARS-CoV-2-specific spike protein pseudotyped lentivirus particles (spike virus) or particles without a viral envelope (Δ spike). A group of cells inoculated with spike virus or Δ spike was left untreated. At 48 h postinoculation, pseudotype virus entry was analyzed by luminescence readout (normalization against untreated spike virus entry). Data represent the mean ± SEM from three independent experiments carried out in technical triplicates. Unpaired two-tailed *t*-test analysis was used to calculate statistical significance. One biological experiment of Otamixaban was excluded due to technical error. Concentrations are displayed in logarithmic scale and molar concentrations. The logarithmic scale is used for better visibility of the wide range of concentrations that are used in these assays.

A total of nine compounds selected from the virtual screening (five) and literature search (four) (Table 1) were tested in a SARS-CoV-2 pseudovirus assay to measure inhibition of viral cell entry, as described by Walls et al. [24]. For this initial in vitro screening, we tested the inhibitory capacity of selected candidates across six different concentrations (Figure 2).

Our cell-based assay revealed a dose-dependent inhibitory effect of the positive control apilimod, thus confirming an appropriate experimental setup. In addition to apilimod, we found two more candidates that show dose-dependent inhibition of virus infection—entrectinib and nilotinib. Both compounds reduce viral infection at their highest concentrations of 25% (entrectinib) and 38% (nilotinib). Furthermore, no activity was found for argatroban, otamixaban, or ivermectin—compounds that had been related to anti-SARS-CoV-2 viral activity in the literature [19,20]. Finally, the effect of the isolated compounds on the cell viability was assessed with a standard MTT assay and confirmed that the observed decrease in luciferase signal is not the result of affected cell viability (see Section 4.7 (Pseudovirus Assay)).

#### 2.2.2. Apilimod, Entrectinib, and Nilotinib Inhibit VSV.G Cell Entry

**Figure 3:**
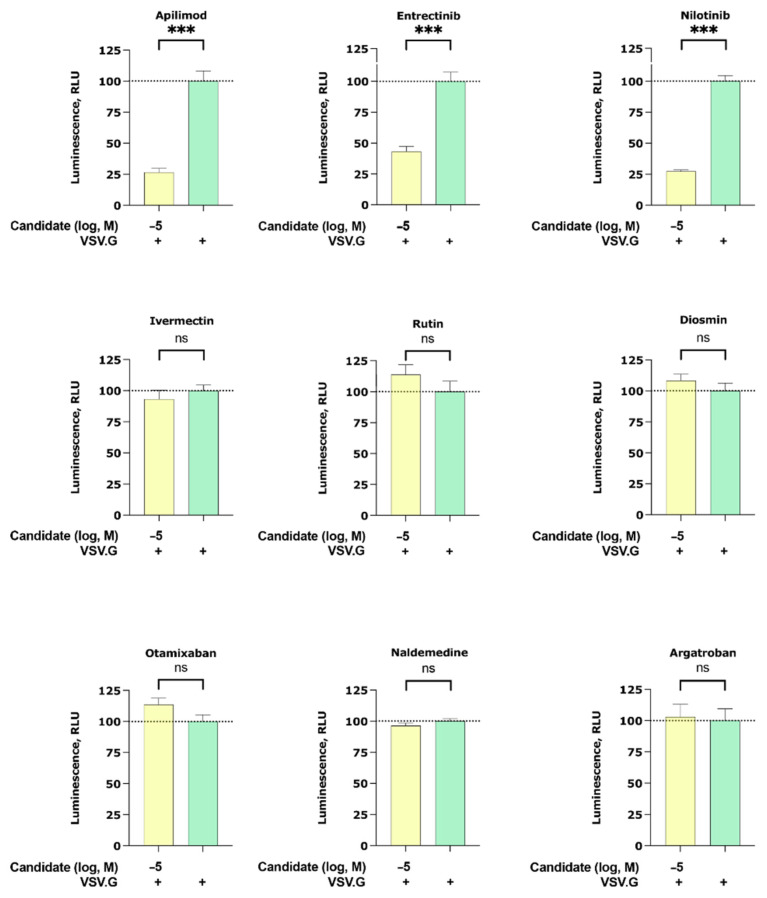
Inhibition of the cell entry of vesicular stomatitis virus G (VSV.G) pseudotyped particles. The 293 T cells transiently expressing human ACE2 and TMPRSS2a were pre-treated with the selected compounds at 10 µM for 3 h before they were inoculated with VSV.G envelope protein pseudotyped lentivirus particles. At 48 h postinoculation, pseudotype virus entry was analyzed by luminescence readout (normalization against untreated VSV.G virus entry). Data represent the mean ± SEM from three independent experiments carried out in technical triplicates. Unpaired two-tailed *t*-test analysis was used to calculate statistical significance (*p* > 0.05 (ns), *p* < 0.001 (***) compared to untreated spike virus entry). Concentrations are displayed in logarithmic scale and molar concentrations. The logarithmic scale is used for better visibility of the wide range of concentrations that are used in these assays.

To interrogate if our candidates can inhibit other enveloped viruses, we studied their inhibitory effect on the cell entry of particles pseudotyped with the membrane protein of vesicular stomatitis virus G (VSV.G). Interestingly, we found that apilimod, entrectinib, and nilotinib were able to partially inhibit VSV.G infection (Figure 3). Note that among the VSV.G active compounds (apilimod, entrectinib, and nilotinib), entrectinib showed the least VSV.G inhibitory while ivermectin, rutin, diosmin, otamixaban, naldemedine, and argatroban did not elicit any activity.

#### 2.2.3. Entrectinib Inhibits Cell Infection in Human Lung Tissue (HLT) Cells at Non-Cytotoxic Concentrations

**Figure 4:**
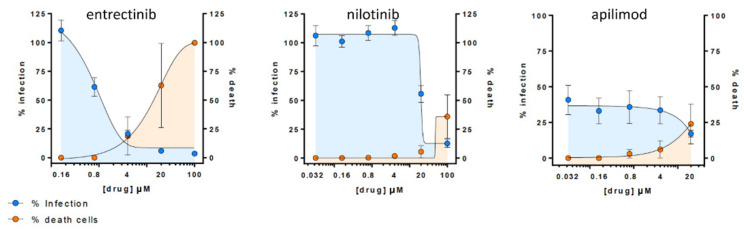
Antiviral assay in Human Lung Tissue (HLT) cells. Percentage of viral entry in HLT cells exposed to VSV*ΔG (Luc)-spike in the presence of the selected compounds. Non-linear fit model with variable response curve from at least two independent experiments in replicates is shown (blue lines). Cytotoxic effect on HLT cells exposed to drug concentrations in the absence of virus is also shown (orange lines).

To validate if the observed in vitro inhibitory effect of detected candidates (i.e., entrectinib and nilotinib) translates into more native conditions, we carried out an antiviral assay in HLT cells [25]. Remarkably, entrectinib exhibited a potent decrease of cellular infection without inducing significant cell death (EC_50_ < 1 µM) (Figure 4). Nilotinib was also found to be active but only at high concentrations (EC_50_ > 15 µM). Interestingly, the positive control apilimod diminished the infection rate to 50% already at very low concentrations, which points to a cytostatic effect of apilimod in this HLT assay. This effect was consistent and confirmed in two repetitive experiments (two different lung donors).

## 3. Discussion

In this study, we used virtual screening to identify potential drug candidates that are able to block SARS-CoV-2 entrance into human cells by targeting the RBD–ACE2 interface. The most promising candidates were tested for their antiviral activities in a SARS-CoV-2 pseudovirus assay. Thereby, we identified two candidates, entrectinib (an inhibitor of tyrosine receptor kinases A/B/C, ROS1, and anaplastic lymphoma kinase [26]) and nilotinib (a Bcr-Abl tyrosine kinase inhibitor [27]), which showed similar viral entry blocking effects in vitro compared to the positive control apilimod (Figure 2).

The observed antiviral SARS-CoV-2 activity of nilotinib is in line with a recent study by Cagno et al. [28] supporting our findings. However, to our knowledge, we are the first ones that report the in vitro SARS-CoV-2 activity of entrectinib. Most importantly, we find that its in vitro antiviral effect translates into HLT cells at non-cytotoxic concentrations (Figure 4), making entrectinib a potential candidate to combat SARS-CoV-2 infections. However, interfering with the RBD–ACE2 binding with small molecules is challenging, and further studies are required to confirm the observed antiviral effect as well as the exact molecular mechanism of its action. It is likely that this mechanism is more complex as entrectinib has been proposed to bind, in addition to ACE2, to different structural and non-structural proteins of SARS-CoV-2 [29]. The same tendency is observed for nilotinib [29]. In addition, nilotinib has been reported to inhibit virus–cell fusion for SARS-CoV and MERS-CoV in vitro [30,31]. Altogether this points to a more complex mechanism of action including likely multiple targets.

Another interesting observation of our study is that entrectinib, nilotinib, and the positive control apilimod are able to partially block cell entrance of another enveloped virus that does not belong to the coronavirus family, the VSV.G (Figure 3). With respect to apilimod’s activity, this effect seems to be only evident at higher apilimod concentration, as VSV.G entry inhibition was not observed by Ou et al. [22] with a concentration of 300 mM. As our experimental setup does not allow concluding about the molecular mechanism of VSV.G inhibition, future studies are required to address the mechanism of action, which can differ for each of the tested compounds.

In conclusion, our study reports antiviral activity of entrectinib against SARS-CoV-2 in human lung tissue, which has not been previously reported. Entrectinib is an FDA-approved drug for the treatment of solid tumors with NTRK fusion proteins and for ROS1-positive non-small cell lung cancers. As an approved drug against lung cancer, it has validated distribution properties, including the lung tissue, upon oral application, which can be beneficial for the treatment of COVID-19. Nevertheless, as an anticancer drug, it can also cause several undesired side effects depending on the dose regime and treatment duration (e.g., fatigue, dizziness, swelling of the legs, and liver toxicity). In this respect, alternative administrations via inhalation can be beneficial as the drug is directly delivered to the target organ, conferring high pulmonary drug concentrations while reducing systemic drug concentrations. Ultimately, further studies are required to confirm these data and the molecular mechanism of action for our best candidate entrectinib.

## 4. Materials and Methods

### 4.1. RBD–ACE2 Interface Examination

To find relevant regions that mediate contacts in the interface between the RBD and the ACE2, a 10 μs simulation of the RBD–ACE2 complex (PDB ID 6VW1) from D.E. Shaw’s laboratory was analyzed (Simulation ID: DESRES-ANTON-10875755) [32]. For that, we used GetContacts 2.0 software [33] to obtain the frequency of total contacts that each interface residue makes during the simulation. Residues with more than 90% contact frequency were considered to be contact hotspots of relevance for virtual screening.

### 4.2. Database Creation

A database of every FDA- and EMA-approved drug was created for the subsequent analyses. For this purpose, DrugBank [34] and PubChem [35] databases were used, merging both of them in a single database in SDF format. Redundancies in both databases were avoided by identifying common IDs. Intermediate drug metabolites were also included from the ZINC15 HMDB Drug Metabolites database [36]. The resulting database included a total of 5849 compounds.

**Figure 5:**
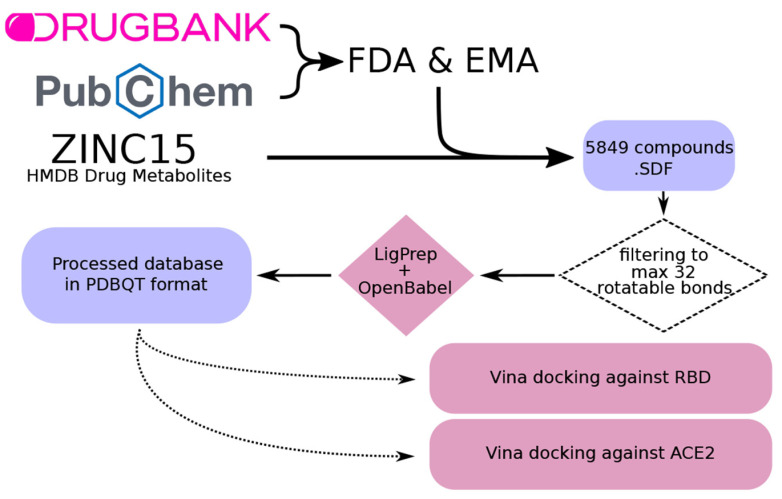
Workflow for the creation of a curated database including drugs approved by the Food and Drug Administration (FDA) and the European Medicines Agency (EMA), as well as known drug metabolites. First, compounds were filtered to discard those with more than 32 rotatable bonds, which are not suitable for docking. Next, different conformations were created using the LigPrep software [39]. Last, conformations were converted into PDBQT format using OpenBabel [40]. The obtained compounds were used for docking against the RBD and ACE2.

Next, we removed compounds that could not be properly docked by the molecular docking software (AutoDock Vina 1.1.2 [37,38]), i.e., >32 rotatable bonds as recommended by the software documentation. Then, different conformations for each compound were found using LigPrep software [39]. Finally, every conformation was converted to PDBQT format using OpenBabel 2.4.0 software [40] (Figure 5).

### 4.3. Molecular Docking

For the molecular docking, AutoDock Vina 1.1.2 software [37,38] was used. First, RBD and ACE2 proteins were prepared and converted into PDBQT format using AutoDock Tools 1.5.6 [41]. Both proteins were obtained, in an unbound state, from a simulation from D.E. Shaw’s laboratory (Simulation ID: DESRES-ANTON-10895671 [32], based on PDB ID 6VW1, replicate 000151, first frame), also available in SCoV2-MD [42]. Then, the docking was performed against the RBD and ACE2, using the previously created database (see Section 4.2 (Database Creation)). The docking region was set to cover the interface contact hotspots detected from the molecular dynamics simulation (see Section 4.1 (RBD–ACE2 Interface Examination)), with its dimensions being 18 Å × 30 Å × 40 Å (see docking box in Figure 1B). This process was made using an exhaustiveness parameter of 10. Top hits were analyzed using VMD 1.9.3 software [43].

Among the docked compounds, five promising candidates were selected based on the energy rankings of the docking against RBD and ACE2, also considering their molecular weight, structure, and published results on their effect on SARS-CoV-2: entrectinib, naldemedine, nilotinib, rutin, and diosmin. Particularly, entrectinib was the highest-scoring compound in the ACE2 screening. Naldemedine was the highest-scoring compound in the RBD screening and among the top hits in the ACE2 screening. Nilotinib was a top hit in the RBD screening, and also it has been proven to inhibit SARS-CoV-2 in vitro [28]. Last, rutin and diosmin were both top-scoring compounds in the ACE2 screening and were selected due to their size (~610 Dalton) and the abundance of hydroxyl groups that could facilitate interface binding.

### 4.4. Plasmids and Cell Lines

The Lenti X 293 T cell line (632180) was purchased from Takara Bio. Third generation lentivirus packaging plasmids—pLenti CMV Puro Luc (17477), pMDLg.pRRE (12251), pRSV.REV (12253), and pMD2.G (12259)—were purchased from Addgene. Human ACE2 plasmid was ordered from Genescript. Human TMPRS2 (pUNO1-hTMPRSS2a) was ordered from InvivoGen. Full-length SARS-CoV-2 Spike plasmid (VG40589-UT) was purchased from Sino Biological. The Spike ORF was subcloned into pcDNA3.1(+) vector via Gibson Assembly (NEB). Subsequently, a truncated version without the C terminal 21 amino acids, the Spike_CTR plasmid, was generated via Q5 Site-Directed Mutagenesis (NEB).

### 4.5. Small Molecules

Small molecules were purchased from MedChemExpress at 10 mM in DMSO.

### 4.6. Pseudovirus Production

On Day 1, Lenti X 293 T cells were seeded at a density of 60,000 cells/cm^2^ per T175 flask in 34 mL DMEM supplemented with 10% FBS and 1 mM sodium pyruvate. On Day 2, plasmid co-transfections were performed with 1 mg/mL PEI MAX (24,765, Polysciences) with DNA:PEI at a ratio of 1:3 as follows. Spike: 20 µg pLenti Luc, 20 µg pMDLg.pRRE, 9.5 µg pRSV.REV and 10.5 µg Spike_CTR at a molar ratio of 1:1:1:0.5, respectively. VSV.G: 20 µg pLenti Luc, 20 µg pMDLg.pRRE, 9.5 µg pRSV.REV and 6.5 µg pMD2.G at a molar ratio of 1:1:1:0.5, respectively. Δ Spike: 20 µg pLenti Luc, 20 µg pMDLg.pRRE, 9.5 µg pRSV.REV at a molar ratio of 1:1:1, respectively. Plasmids and PEI were prepared separately in 1 mL OptiMEM, then combined and incubated at room temperature for 15 min. Then, 2 mL DNA/PEI complex was added to the media and mixed gently. On Day 5, media was collected and sterile filtered into a 50 mL tube via a 0.2 µm syringe filter. Then 5 mL of 3 M NaCl and 10 mL of 50% PEG 8000 (MD2-250-13, Molecular Dimensions) were added to the virus supernatant for a final 0.3 M NaCl and 10% PEG, respectively. The virus was mixed gently by inverting the tube a few times and incubated at 4 C for 24 h. On Day 6, tubes were centrifuged at 4 °C for 45 min at 1500 g, and the resulting pellet was resuspended in 3.5 mL of DMEM + 10% FBS + 1 mM sodium pyruvate. Aliquots of 1.8 mL virus were stored at −80 °C. All virus and assay work was performed in a BSL2 facility. Firefly Luciferase, encoded by pLenti Luc, was used as the assay reporter. VSV.G, encoded by pMD2.G, was used as positive control. Δ Spike was used as negative control.

### 4.7. Pseudovirus Assay

On Day 1293, T cells were seeded at 30,000 per well in white solid bottom 96 well plates that were either poly-L-lysine or collagen coated in completed media (DMEM + 10% FBS + 1 mM sodium pyruvate) at 37 °C and 5% CO_2_. On Day 2, cells were transfected with 150 ng of hACE2 + 15 ng hTMPRSS2a per well with lipofectamine 2000 in OptiMEM according to manufacturer instructions. At 6–8 h post-transfection, the transfection media was replaced with 100 µL DMEM + 10% FBS. On Day 3, cells were incubated with 20 µL of 6× concentrations in HBSS of either the small molecules/peptides or an anti ACE2 antibody (AG-20A-0037PF-C500, Adipogen) for three hours. Subsequently, frozen virus aliquots were thawed, and 100 µL of virus solution was added to the respective wells. On Day 5, the assay plates were equilibrated to room temperature, and the cells were washed once with 200 µL sterile PBS and incubated with 100 µL of substrate (50 µL PBS + 50 µL ONE-Glo EX substrate, E8130-Promega) in the dark for 10 min. The luciferase signal was measured on a PHERAstar plate reader (BMG Labtech).

In order to achieve an optimal transfection efficiency, the ratio and amount of ACE2 and TMPRSS2a DNA was titrated, from 10 ng to 200 ng per well. The best signal to background upon transduction with Spike pseudo virus was achieved with a ratio of 1:10 of TMPRSS2a:ACE2 at 15 ng and 150 ng, respectively.

**Figure 6:**
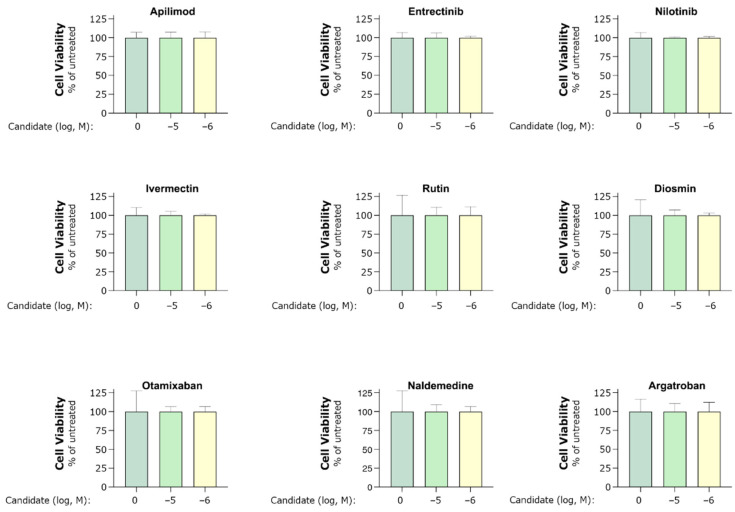
MTS assay of cell viability indicated by mitochondrial activity. HEK293 cells co-transfected with hACE2 and hTMPRSS2a were treated with 1 and 10 µM of candidate for 40–44 h. Assays were performed according to the manufacturer’s instructions. The mitochondrial activity in untreated cells was assigned a value of 100. Experiments were performed in one to three biological replicates and technical triplicates. Bars indicate mean ± standard deviation.

Cell viability (Figure 6) was assessed using the CellTiter 96 Aqueous One Solution (G3582, Promega) according to the manufacturer’s instructions. In brief, cells were incubated in 20 µL of the CellTiter solution and incubated for 4 h before measuring absorbance at 490 nm on a Flex Station plate reader (Molecular Devices). All data were analyzed using the GraphPad Prism software 8.

### 4.8. Antiviral Assays in Human Lung Tissue (HLT) Cells

The antiviral assays were performed as recently described [25]. Briefly, non-neoplastic areas of lung tissues were obtained from patients undergoing thoracic surgical resection at the Thoracic Surgery Service of the Vall d’Hebron University Hospital. The study protocol was approved by the Ethical Committee (Institutional Review Board number PR(AG)212/2020). Tissue was enzymatically digested with 5 mg/mL collagenase IV (Gibco) and 100 µg/mL of DNase I (Roche) for 30 min at 37 °C and 400 rpm and mechanically digested with a pestle. The resulting cellular suspension was washed twice with PBS and resuspended in fresh medium (RPMI 1640 supplemented with 5% FBS, 100 U/mL penicillin, and 100 µg/mL streptomycin) and DNase I to dissolve cell aggregates. Cell number and viability were assessed with LUNA™ Automated Cell Counter (Logos Biosystems). Triplicates of five-fold serial dilutions of the antiviral compounds were tested in HLT cells using 2–3 different donors. Drug dilutions were prepared in R10 in a 96-well plate. HLT cells were added at a density of 300,000 cells/well and incubated with the compound for at least 1 h before infection. Then, multiplicity of infection (MOI) 0.1 of VSV*ΔG(Luc)-S virus, generated as previously described with the mutation D614G and a deletion in the last 19 amino acids in the spike (plasmid kindly provided by Javier García-Pérez, Instituto de Salut Carlos III, Spain) were added to the plates and spinoculated at 1200 g and 37 °C for 2 h. Cells were then cultured overnight at 37 °C in a 5% CO_2_ incubator. Subsequently, cells were incubated with Britelite plus reagent (Britelite plus kit; PerkinElmer) and transferred to an opaque black plate. Luminescence was immediately recorded by a luminescence plate reader (LUMIstar Omega). To evaluate cytotoxicity, we used the CellTiter-Glo^®^ Luminescent kit (Promega), following the manufacturer’s instructions. Data were normalized to the mock-infected control, after which EC_50_ and CC_50_ values were calculated using Graph-Pad Prism 7.

## Figures and Tables

**Table 1:** Compounds selected for the in vitro inhibition activity assay based on docking or on evidence in the literature.

Compound Name	Source	Docking Assay
Nilotinib	Virtual screening	RBD docking
Entrectinib	Virtual screening	ACE2 docking
Rutin	Virtual screening	ACE2 docking
Diosmin	Virtual screening	ACE2 docking
Naldemedine	Virtual screening	RBD and ACE2 docking
Apilimod	Literature [18] (used as positive control in our validation experiments)	-
Argatroban	Literature [20]	-
Otamixaban	Literature [20]	-
Ivermectin	Literature [19]	-

## Data Availability

The data presented in this study are available on request from the corresponding author.
